# Advancing the immunoaffinity platform AFFIRM to targeted measurements of proteins in serum in the pg/ml range

**DOI:** 10.1371/journal.pone.0189116

**Published:** 2018-02-13

**Authors:** Anna Säll, Daniel Corbee, Sara Vikström, Filip Ottosson, Helena Persson, Sofia Waldemarson

**Affiliations:** Department of Immunotechnology, Lund University, Lund, Sweden; Consiglio Nazionale delle Ricerche, ITALY

## Abstract

There is a great need for targeted protein assays with the capacity of sensitive measurements in complex samples such as plasma or serum, not the least for clinical purposes. Proteomics keeps generating hundreds of biomarker candidates that need to be transferred towards true clinical application through targeted verification studies and towards clinically applicable analysis formats. The immunoaffinity assay AFFIRM (AFFInity sRM) combines the sensitivity of recombinant single chain antibodies (scFv) for targeted protein enrichment with a specific mass spectrometry readout through selected reaction monitoring (SRM) in an automated workflow. Here we demonstrate a 100 times improved detection capacity of the assay down to pg/ml range through the use of oriented antibody immobilization to magnetic beads. This was achieved using biotin-tagged scFv coupled to streptavidin coated magnetic beads, or utilizing the FLAG tag for coupling to anti-FLAG antibody coated magnetic beads. An improved multiplexing capacity with an 11-plex setup was also demonstrated compared to a previous 3-plex setup, which is of great importance for the analysis of panels of biomarker targets.

## Introduction

The human proteome is greatly affected during disease and is therefore a rich source of potential protein biomarkers for disease diagnostics. The human plasma proteome reflects both physiological and pathological processes and has been characterized as the most extensive human proteome [1]. Plasma is the preferred clinical sample format because of its low invasive sampling. However, due to the wide dynamic range of protein concentrations and its great complexity, detection of low abundant target proteins from human plasma or serum is challenging. Several thousands of proteins are predicted to be present at low concentrations, potentially a rich source of biomarkers for novel diagnostics and prognostics [2,3] [4,5]. Mass spectrometry (MS) has been instrumental for the discovery of novel potential protein biomarkers, while immunoassays, such as of ELISA, dominate the validation state [6]. Targeted MS through selected reaction monitoring (SRM) provides highly specific and accurate detection and quantification [7,8]. By combining SRM for readout specificity and affinity enrichment using antibodies for increased sensitivity, different technology platforms have been established that have proven suitable for detection of target proteins in complex biological samples [6,9–11].

The quality of antibodies is essential for increasing both the sensitivity and efficacy for the identification of target molecules in immunodiagnostics [12]. Polyclonal antibodies has been dominating the field [5,9,10]. However, the use of monoclonal antibodies has more recently increased [13,14] and offers great advantages due to the renewable capacity and specificity profile. Still, the development of monoclonal antibodies based on hybridoma technology is tedious and costly and remains a major bottleneck in the generation of immunoaffinity-SRM assays. Recently, it has been shown that recombinant antibody fragments, such as single chain variable fragments (scFv) or fragment antigen-binding (Fab), generated from large antibody libraries are well suited as affinity reagents in affinity SRM [11,15]. Recombinant antibody fragments offer a renewable source that are easily produced in bacteria. Other advantages include a higher control over the development process, thereby allowing the generation of antibody fragments with different characteristics [16–18]. The direct knowledge of the antibody gene sequence also allows for convenient transfer into molecular formats to fit the intended application.

Antibodies utilized as affinity reagents for capture of target proteins are immobilized onto a solid support. It is of outmost importance to immobilize the antibody so that it retains its biological activity and that the antigen-binding site is properly exposed and available for binding. Direct covalent coupling is usually performed with a chemical reaction between functional reactive groups of the solid support and free amine/carboxyl groups of the antibody [6]. The outcome is an over-time stable immobilization. This however provides a random antibody immobilization that may risk blocking of their antigen-binding sites [2]. There is also a risk of the antibody being denatured due to strain from attachment at more than one site and that neighboring antibodies can cause steric hindrance [19]. Random covalent coupling also results in a heterogenic immobilized layer of antibodies [20,21]. Alternatively, full-length antibodies can be coupled in an oriented manner by utilizing an intermediate antibody binding protein, such as Protein A or G [22]. For recombinant antibody fragments, C- or N-terminal located affinity tags, can be exploited for an oriented immobilization [20]. By controlling the orientation of the attachment, the antibody antigen-binding site is exposed outwards enhancing its accessibility to the antigen and establishing a more homogenous immobilized antibody layer. A non-covalent coupling also allows for antigen capture in-solution allowing free scFv and target protein to first interact before addition of the magnetic beads. This potentially provides a more efficient antibody-antigen interaction by alleviating the negative effects associated with a random covalent coupling approach [23]. Effective antibody-antigen interaction also depends on the immobilized surface density. There needs to be a high enough surface density to capture enough target protein, although, not too high in order to avoid steric hindrance [6,9–11]. Recombinant antibody fragments allow, through their small molecular size, an increase in immobilization density and thereby also an increased capacity of the solid support [21,24].

**Fig 1:**
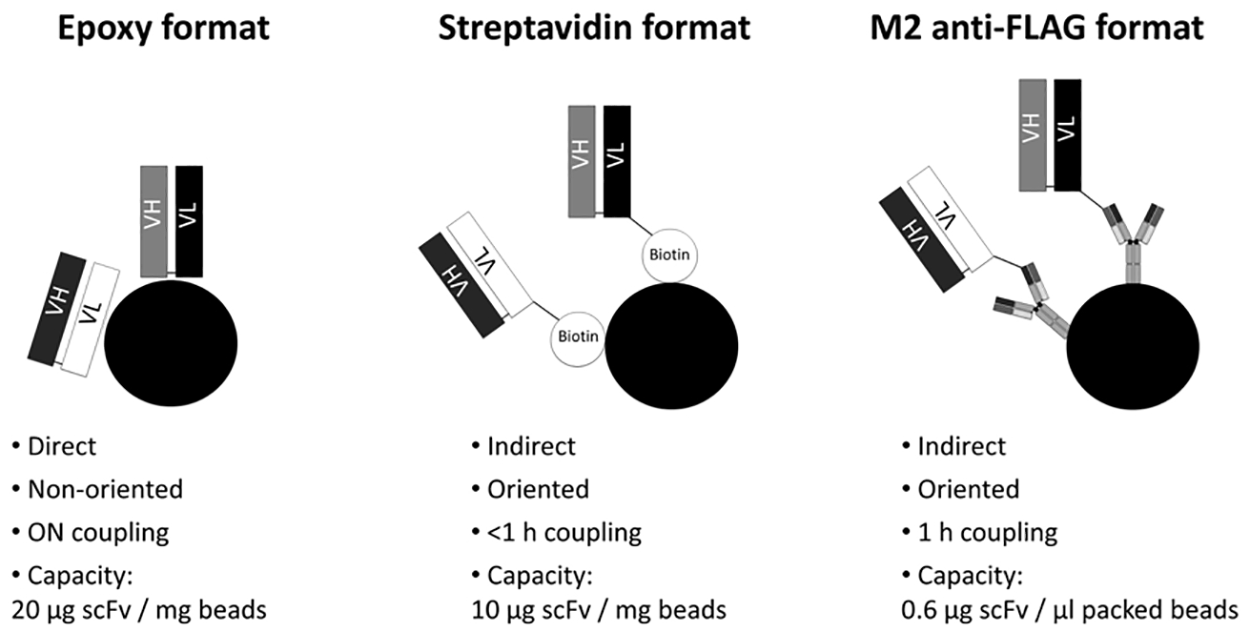
Schematic figure and features describing the three different scFv magnetic bead coupling strategies; epoxy, streptavidin and anti-FLAG. The epoxy format is different to the other two in that scFv are coupled directly to the magnetic beads through a covalent coupling to any lysine residue on the scFv. This results in randomly oriented (non-oriented) scFv on the beads. As opposed to the streptavidin-coated beads and M2 anti-FLAG beads where the scFv is immobilized through coupling to the bead surface through a linker at the back-end of the scFv and thus an oriented coupling that is not interfering with the binding site of the scFv. The steptavidin and anti-FLAG formats also require much less scFv and shorter coupling time.

We have previously developed the AFFIRM platform and demonstrated that immobilized scFv can be used for targeted enrichment of low abundant proteins from complex mixtures such as serum followed by targeted SRM analysis for a specific readout of target peptides in single- and multiplexed assays [5,9–11]. Here, we have further advanced the AFFIRM platform by exploring three different immobilization strategies for coupling of the scFv antibody fragments to magnetic beads (Fig 1). Direct non-oriented immobilization through the use of epoxy-beads and two indirect oriented immobilization strategies through coupling biotin-tagged scFv to streptavidin beads or by exploiting the FLAG-tag for coupling to anti-FLAG antibody coated beads were investigated in parallel in regards to capture and detection of target proteins in both single- and multiplexed assays (11-plex). In addition, the streptavidin-biotin system was used for evaluating the performance of antigen capture in-solution, allowing the scFv-antigen binding to take place in-solution before adding magnetic beads for antigen isolation.

## Materials and methods

### Materials

Eleven target proteins were used in this study. InaD-like protein (INADL-1), calcium/calmodulin-dependent protein kinase type IV (KCC4), alpha-1-syntrophin (SNTA1), serine/threonine-protein kinase (MARK2-1) and interleukin-6 (IL6), casein kinase I isoform epsilon (CSNK1E), tyrosine-protein kinase (FER), receptor-type tyrosine-protein phosphatase T (PTPRT), serine/threonine-protein phosphatase (PGAM5), receptor-type tyrosine-protein phosphatase eta (PTPRJ) and cyclin-G-associated kinase (GAK). All proteins were obtained through the EU funded AFFINOMICS project [25].

### scFv antibody generation and production

ScFv antibodies were generated with phage display selections from the in-house designed and constructed Hell-library as previously described [11,26]. The scFv selected for the target proteins used are listed in S1 Table. Each scFv were produced using two different vector formats. The first format provided the secreted scFv with a triple-FLAG tag followed by a hexahistidine tag at the C-terminus. In the second format, the triple-FLAG tag had been exchanged for a biotin acceptor domain (BAD) previously described [27], allowing for site-specific in vivo biotinylation. The vectors encoding the triple-FLAG constructs were transformed into Top10 *E*.*coli* and scFv produced by cultivating the scFv clones in 15 ml TB medium (Becton, Dickinson and Company, Franklin Lakes, New Jersey, USA) supplemented with 0.2M sucrose and 25 μg/ml kanamycin (Saveen & Werner AB, Sweden). The expression of the scFv fragments was induced by addition of 1 mM isopropyl thiogalactioside (IPTG, Saveen Werner), the scFv clones were grown overnight at 30°C and then harvested by centrifugation. The BAD-constructs were transformed into AVB101 (Avidity LLC, Aurora, Colorado, USA). AVB101 is an *E*. *coli* B strain containing pBirAcm, encoding biotin ligase and chloramphenicol resistance (Avidity LLC, Aurora, Colorado, USA). ScFv antibodies were produced and purified as described above with the exception that 10 μg/ml chloramphenicol (Saveen & Werner AB) was added during the cultivation in addition to 25 μg/ml kanamycin (Saveen & Werner AB, Sweden) as well as the addition of 50 μM biotin (Thermo Fisher) together with IPTG. Both the expression of the scFv fragments and biotin ligase was induced by addition of IPTG. The produced scFv were purified from the periplasmic space using MagneHis^™^ Protein Purification System (Promega Corporation, Madison, WI, USA) and a KingFisher Flex robot (Thermo Fisher). Both the purity and integrity of the scFv were verified with SDS-PAGE.

### Selection of target peptides and transitions

Shotgun MS-MS/MS analysis was used to generate spectral libraries for the recombinant proteins digested in-solution, as well as for monitoring background in the AFFIRM captures. Samples were analyzed on a Thermo LTQ Orbitrap XL mass spectrometer (Thermo Electron, Bremen, Germany) with an Eksigent 2D NanoLC system (Eksigent technologies / SCIEX) upfront equipped and as previously described [11]. The same mobile phases and LC-gradient as described for the SRM analysis below was used. The data was searched against Mascot (http://www.matrixscience.com, version 2.3.01) and results were used to build a spectral library in the SRM management software Skyline [28]. Target proteins were *in-silico* digested in Skyline and peptides identified in the spectral library were searched against a background proteome (SwissProt Human ver. Feb 2014). One to five unique target peptides of interest with four fragments ions were selected. In addition, one peptide common for all scFv’s used in the experiments was selected and peptide for Apolipoprotein B (APOB) representing background signal. The complete transition list is available as S1 Table.

### Coupling of soluble antibody fragments to magnetic beads

Three different bead systems, magnetic Dynabeads M-270 Epoxy beads (Invitrogen Dynal AS, Olso, Norway), magnetic Dynabeads M-270 Streptavidin beads (Invitrogen Dynal AS, Olso, Norway) and anti-FLAG^®^ M2 magnetic beads (Sigma-Aldrich Corp, St. Louis, MO USA) were used for coupling of the purified scFv antibodies and subsequently for capture of target proteins. For all antigens, two scFv were used for capture of the target protein, except for INADL-1, KCC4 and PTPRT where only one scFv per target were used. When coupling two different scFv’s for one target to the magnetic beads, the scFv’s were mixed prior to addition to the beads. The purified scFv’s were coupled to the magnetic beads according to manufactures protocol. Briefly for Dynabeads M-270 Epoxy, 20 μg scFv was coupled per 1 mg beads. Beads were mixed with 0.1 M sodium phosphatase (pH 7.4), scFv and 1 M ammonium sulfate. The mixture was incubated over night at 37°C with mixing (120 rpm). For Dynabeads M-270 Streptavidin, 10 μg scFv was coupled per 1 mg beads. Washed beads were mixed with scFv and incubated for 45 min with gentle mixing in room temperature. For anti-FLAG^®^ M2 magnetic beads, 30 μg scFv was coupled per 100 μl bead solution. Beads were washed and re-suspended in 50 mM Tris-HCl, 150 mM NaCl (pH 7.4) and then mixed with scFv. The mixture was incubated for 1 h in room temperature with gentle mixing. Bead coupling was performed identically for the single-plex and multiplex experiments. For the multiplex captures, the scFv coupled beads for all targets were mixed before use.

### On-bead capture of target proteins

Capture of the spiked target proteins in human serum (serum mix, Sigma) in single- as well as 11-plex assays was performed using a KingFisher Flex magnetic bead processor as previously described [11]. Trypsin (MS sequence grade, Promega) digestion was performed by adding 20 μl of 6.25 ng/μl of trypsin in 50 mM ambic and incubated overnight at 37°C with shake (850 rpm). Target proteins were captured in 10% (v/v) human serum (Sigma-Aldrich Corp, St. Louis, MO USA) diluted in PBS (100 μl diluted to 1 ml) with target proteins at concentrations of 100, 50, 25, 12.5, 5, 1, 0.5, 0.1 and 0.05 ng/ml for the 11-plex assay and at concentrations of 50, 5, 0.5 and 0.05 ng/ml for the single-plex assay. Twenty μl of coupled beads were used for the single-plex assay and 10 μl of coupled beads for each target protein was used for the multiplexed assay, 110 μl of beads in total. The concentration of the three bead systems Epoxy, Streptavidin and FLAG were 0.05 mg/μl, 0.01 mg/μl and 0.33 μl/μl bead in solution respectively.

### In-solution capture of target proteins

The in-solution captures were also carried out using the KingFisherFlex robot. The capture was initiated by adding 2 μg of scFv diluted in PBS to 980 μl of 10% serum in PBS spiked with target protein as above and incubated with scFv for 3h. Following incubation, 0.25 mg of M-270 Dynabeads Streptavidin beads in PBS supplemented with 0.05% (v/v) Tween20 were added to the solution and incubated for 1h. Following incubation, four washing steps were carried out in PBS supplemented with 0.1% (w/v) CHAPS and finally eluted in 0.03% (w/v) CHAPS, 50 mM Ambic. The buffer was removed and samples were digested identical to the on-bead capture digestion as described above.

### Nano-LC-SRM and data analysis

The nano-LC-SRM analysis was performed on an Eksigent 2D NanoLC system (Eksigent technologies /SCIEX) coupled to a Triple Stage Quadrupole Mass spectrometer (TSQ Vantage, San José, CA) equipped as previously described [11]. Mobile phase A was water/0.1% FA (v/v) and mobile phase B was ACN/0.1% FA. 300 nl/min flow rate was used with a gradient of increasing mobile phase B from 3–35% B during 22 min and from 45–90% during 8 min. 8 μl sample or a 4 μl (epoxy multiplex only) sample was injected for LC-SRM analysis for single and multiplex analysis respectively. Between each analytical sample injection, an injection for system wash and equilibration was performed. The TSQ instrument was operated in positive ion mode with a spray voltage of 1.8 kV and an ion capillary temperature of 270°C. Q1 and Q3 were set to unit resolution (0.7 Da) and analysis run with 10 ms dwell time. Data acquisition was done using Xcalibur software (version 2.1). The resulting data was analyzed in Skyline SRM management software manually integrating target peaks. Data from digested recombinant target proteins analyzed with the SRM assay was used as reference runs to identify the target peptide. Integrated peak intensities were exported and the data was plotted in GraphPad (Prism). Linear regression was performed including all data point down to a concentration when linearity would be greatly obscured by including that concentration or provide a linearity below 0.5. No weighting was used. Intensities were plotted versus concentration. Mean intensity and standard deviation was calculated for each peptide and concentration point to calculate percentage coefficient of variation (%CV).

## Results

The affinity SRM platform AFFIRM allows for capture and detection of low abundant target proteins in complex samples such as serum [11]. The platform makes use of recombinant scFv antibodies coupled to magnetic beads for enrichment of target proteins from the complex mixture. The captured targets are washed, on-bead trypsin digested and detected using LC-SRM readout. AFFIRM has previously used scFv antibodies covalently immobilized onto magnetic beads via surface epoxy groups on the magnetic beads and primary amino groups (e.g. lysines) of the scFv antibody. Although stable, the orientation of the immobilized scFv cannot be controlled. In this study, we therefore explored two additional immobilization strategies for the scFv antibodies that allow for controlling the orientation of the attached scFv (Fig 1). Firstly, a vector construct providing the produced scFv antibodies with a C-terminal located triple-FLAG tag was exploited for immobilizing the scFv’s to magnetic beads pre-coupled with the anti-FLAG M2 monoclonal antibody. Secondly, a site-specific biotinylation site was used to immobilize the scFv antibodies onto streptavidin coated magnetic beads. This was done through introducing a vector format that allows for production of soluble scFv antibodies with a C-terminal located biotin acceptor domain (BAD) [27] [29]. With the use of biotin ligase BirA, a biotin molecule is site-directly attached to a lysine within the BAD domain. The three different immobilization strategies are illustrated in Fig 1. In addition, in order to investigate whether antigen capture levels would improve when free from potential steric hindrance from on-bead coupling, the capture ability of biotinylated scFv in solution, rather than pre-coupled to beads, was evaluated. Furthermore, the multiplexing ability of the platform was challenged by creating immuno-affinity SRM assays for eleven new targets with the use of one to two scFv antibodies per target (S1 Table). The capture and detection of these eleven target proteins were evaluated in the AFFIRM assay workflow with regards to the immobilization strategy in a multiplex (11-plex) assay format as well as in a single-plex format for four of the targets. The concentration range explored was expanded to range from 100 ng/ml down to 50 pg/ml of target protein in serum background. For practical reasons, a concentration of 10% serum was used as background. This was based on an experiment where the same concentration (50 ng/ml) of target protein was spiked in 10, 20, 50 and 80% plasma as well as in 10% serum. The antigen was retrieved to the same degree in all experiments (i.e. output signal in the same range, S6 Fig)). We therefore concluded 10% serum to be a good working concentration for our experiments.

### Single-plex AFFIRM assays

**Fig 2:**
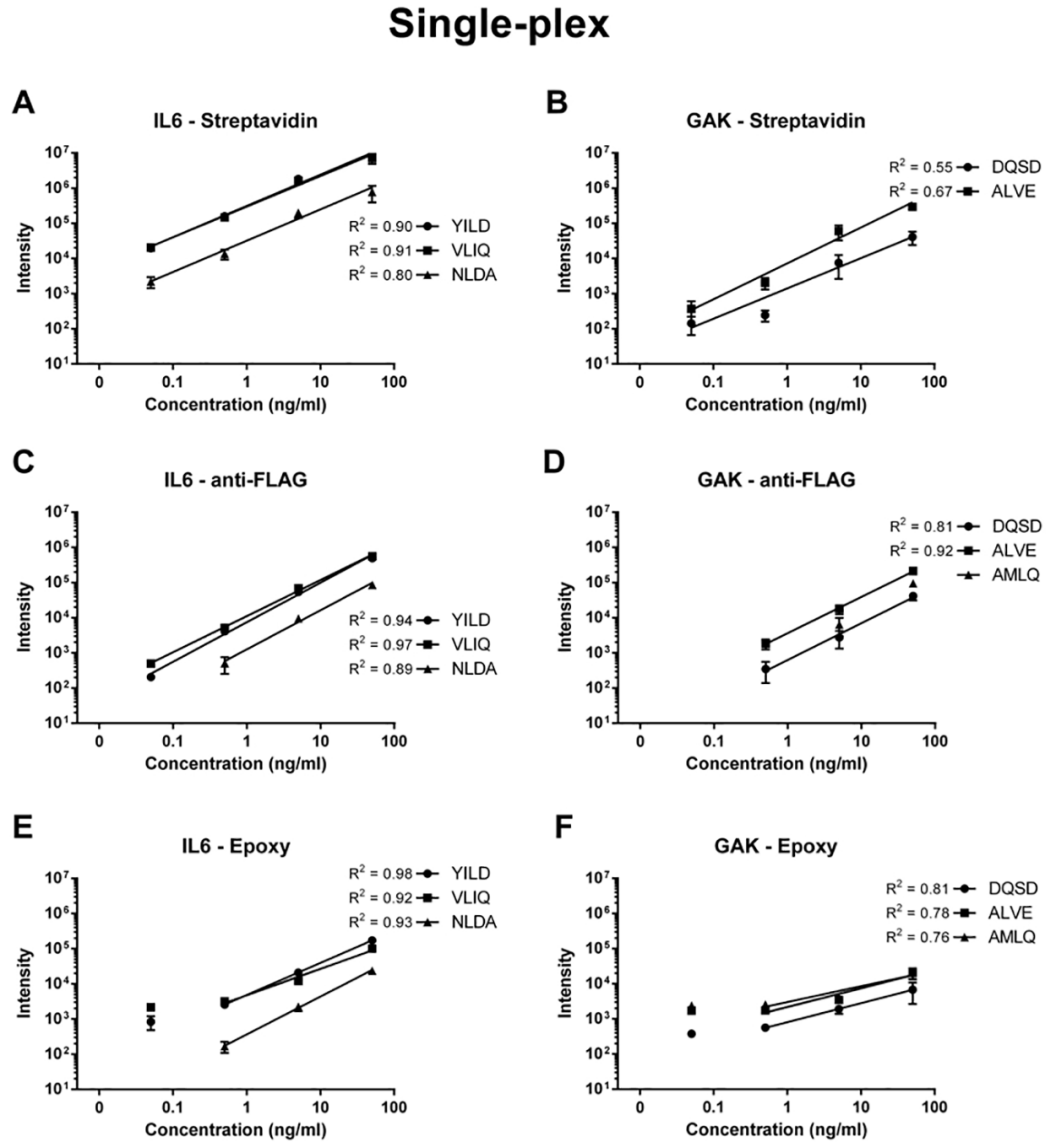
Single-plex captures of target proteins IL6 and GAK in the streptavidin (a, b) anti-FLAG (c, d) and epoxy (e, f) scFv—Magnetic bead coupling system. Target proteins were enriched for from serum background at spike levels of 50, 5, 0.5 ng/ml and 50 pg/ml. Each concentration point was run in triplicates.

Four target proteins, CSNK1E, FER, GAK and IL6, were selected for generating response curves in a single-plex fashion i.e. each protein was enriched for from different samples. Two different scFv per target protein were mixed before coupling to respective bead systems and used for AFFIRM analysis of target proteins spiked at 50, 5, 0.5 ng/ml and 50 pg/ml of target protein in 10% serum in triplicates for each concentration. Response curves from the SRM readout for respective target protein were generated. Response curves for target proteins IL6 and GAK for the three bead systems epoxy, streptavidin and anti-FLAG are displayed in Fig 2. Target proteins CSNK1E and FER did not generate any successful dilution curves and were only detected at a few of the higher concentrations. Captures utilizing the streptavidin immobilization format obtained the highest measured intensities for target proteins IL6 (Fig 2a) and GAK (Fig 2b) and allowed detection of the proteins down to 50 pg/ml. Linear response R^2^ = 0.91 was achieved for IL6 while GAK achieved a poorer linearity (R^2^ = 0.67). While displaying slightly lower intensities than streptavidin, the anti-FLAG immobilization format achieved higher R^2^ for IL6 (Fig 2c) and GAK (Fig 2d) (0.97 and 0.92 respectively) but was unable to measure target protein GAK at the lowest concentration. The AFFIRM captures using the epoxy immobilization format overall displayed the lowest measured intensities of the two target proteins. This system was able to enrich the proteins at all concentrations, however, not with a linear response at the lowest concentration of 50 pg/ml (R2 = 0.98 and 0.81 for IL6 and GAK respectively for concentrations down to 0.5 ng/ml) (Fig 2e and 2f respectively). In conclusion, the two oriented immobilization strategies utilizing streptavidin and anti-FLAG beads displayed the best overall performance for the single-plex capture experiments.

### 11-plex AFFIRM assays

**Fig 3:**
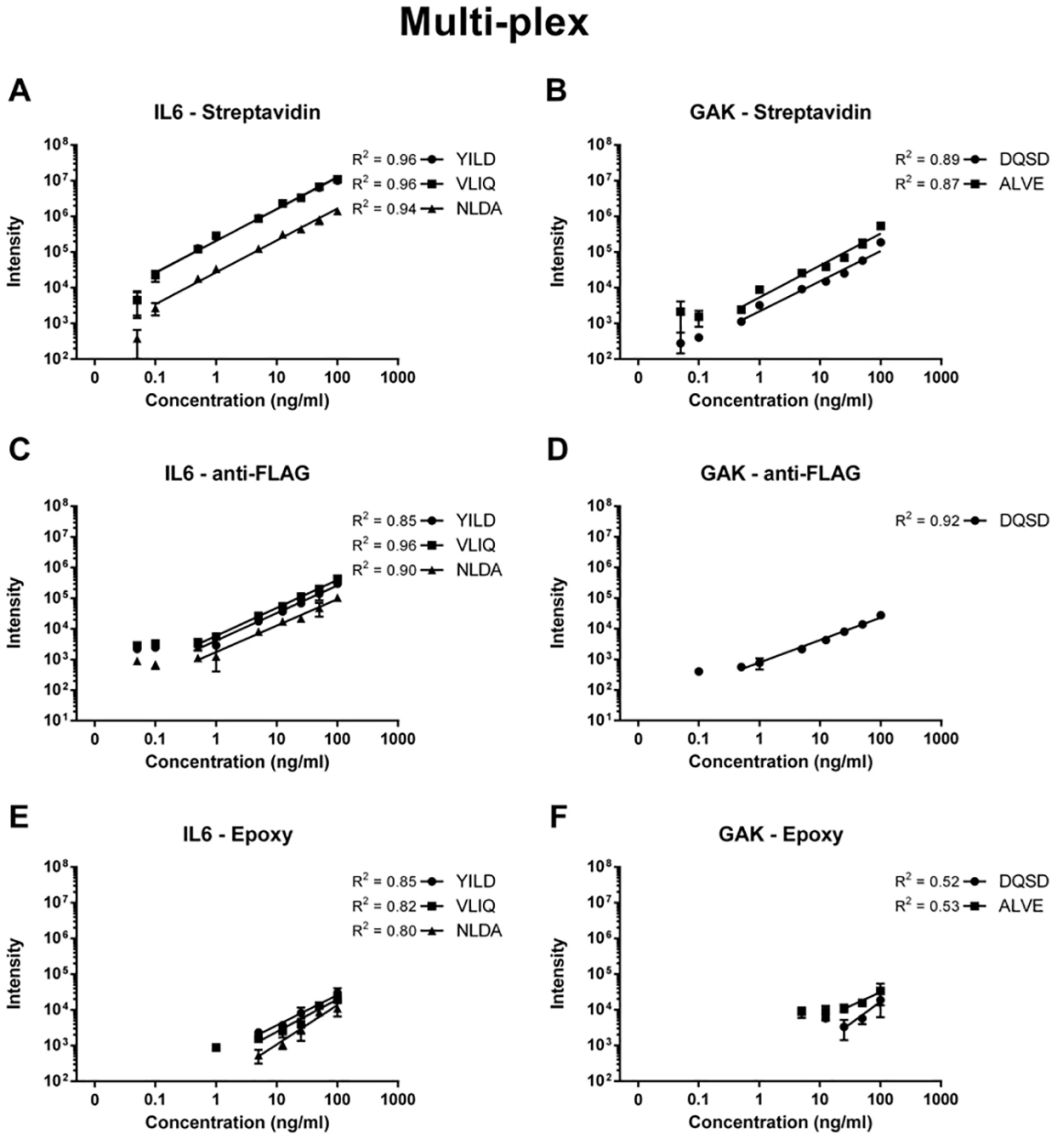
Multiplexed (11-plex) captures of target proteins IL6 and GAK using the streptavidin (a, b) anti-FLAG (c, d) and epoxy (e, f) scFv—Magnetic bead coupling system. Target proteins were enriched for from serum background at spike levels of 100, 50, 25, 12.5, 5, 1, 0.5, 0.1 ng/ml and 50 pg/ml. Each concentration point was run in triplicates.

In previous AFFIRM work, the multiplexing capacity of the platform was demonstrated through targeted enrichment of three different target proteins from one sample [11]. Here we further challenged the platform and evaluated multiplexing through capture of eleven targets from one sample. scFv antibodies, one or two per target, were coupled to the different magnetic bead systems individually for each target protein. Subsequently, magnetic beads for all targets were mixed at equal proportions and used for enrichment of the eleven targets from one sample for each magnetic bead system. The target proteins were captured at concentrations 100, 50, 25, 12.5, 5, 1, 0.5, 0.1 ng/ml and 50 pg/ml spiked in 10% serum. Each concentration point was performed in triplicates. Fig 3 displays response curves for target proteins IL6 and GAK for the respective capture format. Response curves for the remaining nine proteins are provided as S1, S2 and S3 Figs. As also seen in the single captures, the streptavidin system offered the highest measured intensities in the 11-plex captures with good linearity down to 100 pg/ml for IL6 and 0.5 ng/ml for GAK (0.96 and 0.89 respectively) (Fig 3a and 3b). Signals for both proteins were still detected at the lowest concentration of 50 pg/ml however not with linear response. The anti-FLAG system enabled detection of target proteins IL6 at 50 pg/ml (Fig 3c) and GAK (Fig 3d) at 100 pg/ml with good linearity down to a concentration of 0.5 ng/ml (0.96 and 0.92, respectively). As for the single captures, the epoxy immobilization system demonstrated the lowest measured intensities for both target proteins in the multiplexed captures. Protein IL6 (Fig 3e) was measured down to 1 ng/ml with a relatively linear response down to 5 ng/ml (R^2^ = 0.85). Target protein GAK (Fig 3f) was measured at 5 ng/ml but with poor linearity (R^2^ down to 25 ng/ml = 0.53).

**Fig 4:**
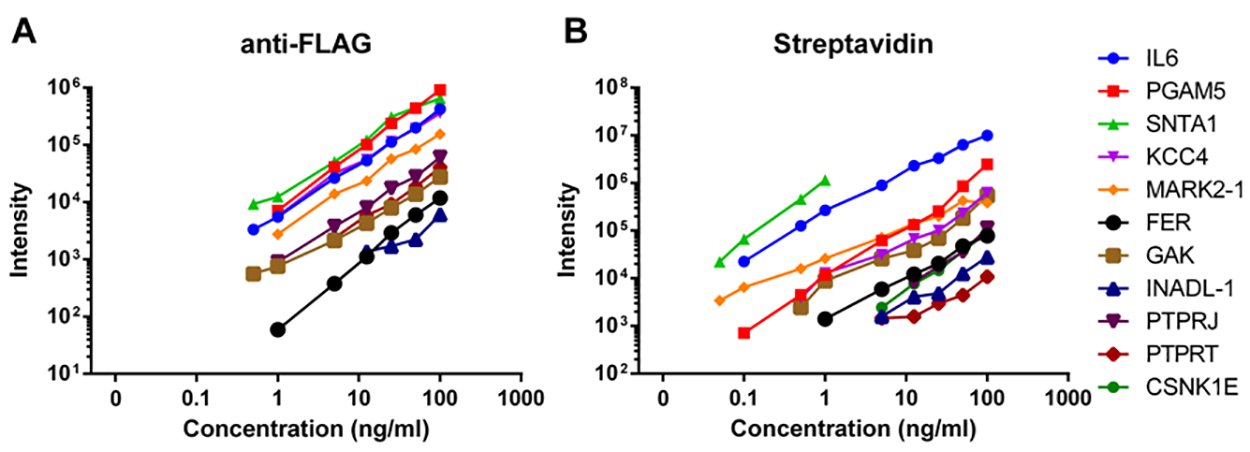
Linear response range for one peptide per target protein in the11-plex AFFIRM experiment in a) the anti-FLAG format and b) the streptavidin format, demonstrating detection with linear response down to low ng/ml concentrations for the majority out of the target proteins.

Overall, the measurements of all eleven target proteins of the assay share the same pattern characteristic of the three bead systems (S1, S2 and S3 Figs). The streptavidin immobilization system offers the highest measured intensities with good linearity allowing most proteins to be measured in the low ng/ml range and allowed for identification of all eleven target proteins (S1 Fig). The anti-FLAG format demonstrates slightly lower intensities than streptavidin but with similar R^2^ values and detection of ten out of the eleven target proteins in the low ng/ml range (S2 Fig). The Epoxy immobilization system falls short in the 11-plex capture experiments as only six of eleven target proteins could be detected and with low overall measured intensity (S3 Fig). An overview of the linear response ranges for all proteins in the 11-plex assays are displayed in Fig 4 for the anti-FLAG (Fig 4a) and streptavidin systems (Fig 4b). High quality AFFIRM assays down to low ng/ml ranges are provided for virtually all proteins for both systems.

In order to assess the overall quality of the multiplexed assay, assay ratings were implemented to provide an overview of how well each target is detected in the different capture formats. The assay ratings, displayed in Table 1, describes how well each measured protein in the multiplexed assay has been determined based on lowest concentration within the linear response range weighted together with the R^2^ value of the linear regression. An A rating has a linear response with R^2^ > 0.9 down to a concentration of 0.05–1 ng/ml, B rated assays have a linear response down to 5–12.5 ng/ml and C rating is everything from 25 ng/ml and above. A linear regression R^2^ value below 0.9 downgrades the assay quality one level (i.e. from A to B or B to C). The streptavidin and anti-FLAG capture formats had 4 and 5 assays rated as A respectively, and 3 and 4 proteins rated as B assays respectively demonstrating very high quality AFFIRM assays for a total of 8 out of 11 proteins targeted. The remaining three proteins could be measured with both the streptavidin and anti-FLAG system at relatively low concentrations, but with poorer linearity (Table 1 and S2 and S3 Figs). Epoxy captures provided no A rated assays and could only measure 6 proteins in total with B rated assays for two of the target proteins (Table 1 and S4 Fig). Table 1 also provides the average coefficient of variation (CV), expressed as the percent relative standard deviation (standard deviation divided by the mean) over the linear range for respective dilution curve. Seven out of the 11 target proteins demonstrate CVs below or close to below 15% in the streptavidin system. The anti-FLAG system provided slightly higher CVs and the epoxy system could only provide a good CV for one of the proteins measured. A complete list of CVs calculated for each concentration point within the linear range and the range averages, is provided in S3 Table.

**Table 1:** Table listing the lowest detection level within the linear response range for one peptide per protein in the 11-plex AFFIRM assay in the three different magnetic bead systems. The assay for each target protein in the three systems is rated into three grades of assays, A-C. Rating is based on lowest detected concentration in the linear response range where linear response is defined by an R^2^ value above 0.9. The assay grades are defined as; A = 0.05–1 ng/ml; B = 5–12.5 ng/ml; C = 25+ ng/ml of lowest detected peptide level in the linear response range. This detection level is combined with the R^2^ value where the assay grade is downgraded one level (i.e. from A to B or from B to C) if the R^2^ value is below 0.9. The lowest concentration of detected target proteins within the linear response is displayed as both ng/ml and fmol/ml for respective bead system. The epoxy system could only detect 6 out of the 11 proteins.

Protein	Streptavidin				anti-FLAG				Epoxy			
	Assay Rating	Lowest protein spike concentration detected (ng/ml)	Theoretical lowest amount of protein detected (fmol)	Mean CV (%)	Assay Rating	Lowest protein spike concentration detected (ng/ml)	Theoretical lowest amount of protein detected (fmol)	Mean CV (%)	Assay Rating	Lowest protein spike concentration detected (ng/ml)	Theoretical lowest amount of protein detected (fmol)	Mean CV (%)
**IL6**	A	0.1	4.2	13.2	B	0.5	21	32.6	C	5	210	25.3
**PGAM5**	A	0.1	3.1	15.9	A	1	31	25.4	B	12.5	390	17.0
**CSNK1E**	B	5	110	31.1	-	-	-	-	-	-	-	-
**SNTA1**	B	0.05	0.93	15.4	B	0.5	9.3	32.4	-	-	-	-
**KCC4**	A	0.5	9.6	17.1	A	1	19	22.8	C	12.5	240	52.3
**MARK2-1**	B	0.05	0.57	39.5	B	1	11	35.6	B	1	11	24.4
**FER**	A	1	11	11.7	A	1	11	19.4	-	-	-	-
**GAK**	B	0.5	3.5	15.5	A	0.5	3.5	17.4	C	12.5	87	69.1
**INADL-1**	C	5	25	15.4	C	12.5	64	47.1	-	-	-	-
**PTPRJ**	C	12.5	86	27.6	A	1	6.9	25.9	-	-	-	-
**PTPRT**	C	5	31	21.3	C	5	31	27.7	C	12.5	77	38.3

The signal intensity of the peptide common to all scFv provides a relative amount of scFv coupled to the beads. Comparing the multiplexed and single captures of IL6 and GAK in the three different bead systems, an increased signal of the scFv peptide for the multiplexed captures compared to single captures is seen, as would be expected from the larger amount of beads used in these experiments (S4 Fig). The epoxy immobilization format displays the highest intensities followed by anti-FLAG format and with streptavidin format showing the lowest signal. This is in accordance with the both the amount of coupled scFv’s and with the amount of beads used for each bead system.

In summary, the use of an oriented immobilization strategy, as here exemplified by the biotin/streptavidin and FLAG-tag/anti-FLAG antibody systems, appears to not only provide higher signal intensities and more linear responses but to also to increase the multiplexing ability of the AFFIRM assay.

### On-bead versus in-solution AFFIRM assays

**Fig 5:**
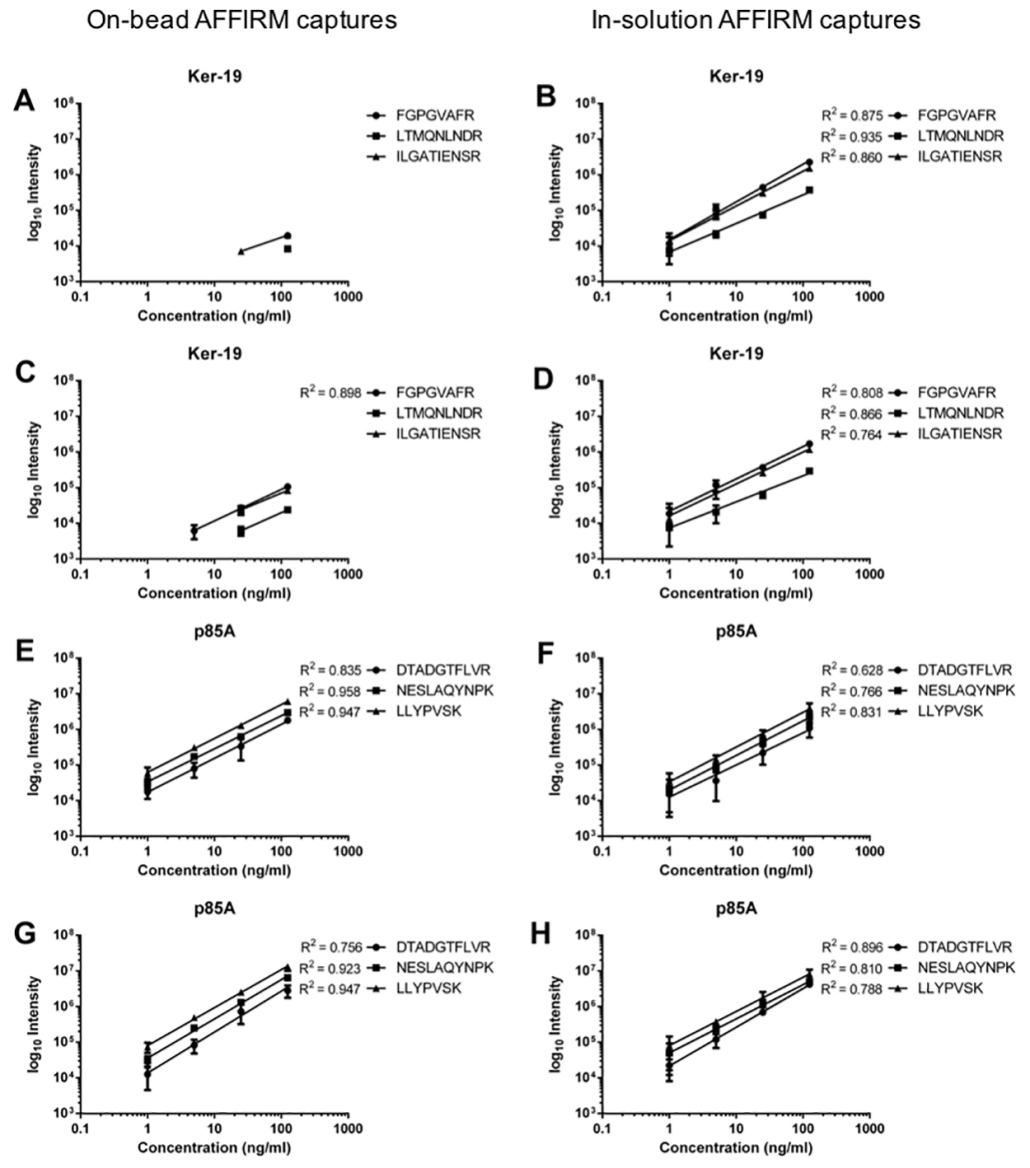
Single-plex AFFIRM captures of target proteins KER19 and P85A performed on-bead (left) according to the standard protocol and in-solution (right) using the streptavidin system. The in-solution captures are allowing antibody and antigen to bind before adding magnetic beads for isolation. Target protein was spiked in serum background from 125 down to 1 ng/ml concentration. Two scFv were used for capture of each protein.

The introduction of directed scFv coupling to the magnetic beads through the use of specific tags also allows for antibody-antigen interaction in-solution before subsequent addition of magnetic beads. This setup, referred to as in-solution captures, was evaluated in parallel with the standard on-bead AFFIRM setup. Two target proteins, KER19 and P85A previously explored in the AFFIRM platform were used and two scFv per target protein were evaluated in single-plex assays. The SRM assay applied in this experiment is provided in S4 Table. The on-bead versus in-solution setups were evaluated performing triplicate AFFIRM analysis of target proteins spiked at 125 ng/ml down to 1 ng/ml concentrations in serum background (Fig 5). The in-solution captures demonstrated better results for KER19 detecting the target protein down to 1 ng/ml while P85A was detected to the lowest concentration in both setups. However, a somewhat better linearity was observed for on-bead capture. Taken together, the in-solution capture setup provides an attractive alternative especially for proteins proven difficult to capture on-bead. The amount of scFv detected in the on-bead setup was as expected always higher than in the in-solution setup (S5 Fig).

## Discussion

Modern medicine will require an increasing number of biomarkers implemented in the clinics for disease diagnosis and stratification, as companion diagnostics for correct choice of treatment regimes as well as for drug development. Large numbers of potential protein biomarker candidates are easily discovered with technologies such as mass spectrometry. However, very few novel biomarkers are approved for clinical use and the lack of suitable technologies for validation remains a major bottleneck [4,10,30]. To meet the need for technology to measure target proteins in low concentrations in body fluids such as plasma or serum reproducibly with through-put we have developed the immunoaffinity platform AFFIRM, which combines the sensitivity of scFv antibodies with the specificity of LC-SRM readout [11]. SRM-MS has for decades been the gold standard technology for measuring small molecules in clinical settings and is increasingly being accepted as attractive for also quantifying peptides with assay qualities of clinical standard [7,31,32]. The possibility of producing scFv antibodies and targeted SRM assays to measure virtually any proteins or combinations thereof make AFFIRM a flexible tool to use in a plug-and-play fashion.

In this study, the detection range of the AFFIRM assay was challenged and by making use of oriented immobilization approach of the scFv to the magnetic beads we could successfully demonstrate 100 times improved sensitivity. We could successfully develop AFFIRM assays for all selected target proteins in the streptavidin system in the multiplexed format without any pre-optimization or selecting targets/scFv particularly successful in the AFFIRM format. This is promising as the lead time to develop AFFIRM assays towards novel proteins is short. The single-plex format was a starting- and reference point for the development of the multiplexed setup and we therefore did not further analyze why two out of the four single-plex experiments failed. The indirect coupling also enables performing captures in-solution, i.e. allowing the scFv and target protein to interact freely in solution before adding magnetic beads for retaining the complex.

Both the anti-FLAG and the streptavidin system detected the target proteins at low ng/ml to pg/ml concentrations with satisfactory reproducibility. The streptavidin system provided superior overall performance with CVs below or close to <15% for many peptides. As a CV of 15% generally is stated as a requirement for clinical assays, this is well in range for biomarker verification purposes where higher CVs may be acceptable [4].

In order to be able to monitor the linear response of the target proteins, a majority of the proteins included in this study were selected to be low or not present in serum. Of the eleven target proteins, only KCC4 and PTPRJ have previously been detected in plasma using MS where KCC4 is present in very low concentration and PTPRJ at higher concentrations (PAX DB–data not shown). No endogenous protein was detected in our experiments as seen by the linearity of the curves down to the lowest concentration measured [33].

The sensitivity of the AFFIRM assay is dependent on not only the orientation of the immobilized antibodies but also the surface density of the attached antibodies. In the epoxy system there is a risk of immobilizing too much antibody that may lower the sensitivity due to steric hindrance and crowding effects impairing antibody-antigen binding [21]. In addition, too much antibody may limit assay sensitivity by the high concentrations of scFv peptide in the final sample. This limits the sample amount feasible to inject for MS analysis without clogging the LC-system. An increase of scFv peptides in the samples was indeed confirmed by providing the highest scFv signal readout as compared to the other two systems (S4 Fig).

Heavy isotope labelled standards is required for an absolute quantification of measured targets in mass spectrometry and to establish assay limit of detection (LOD) and limit of quantification (LOQ). The addition of heavy isotope labelled proteins is, although still costly, becoming increasingly feasible [7]. In this study, blank experiments were performed, i.e. AFFIRM analyses of samples with no protein spike confirming that no severe background noise signal appear upon no spike (data provided in Passel).

In summary, this work demonstrates an expansion of the tools used in the AFFIRM assay through the introduction of two oriented scFv immobilization strategies to magnetic beads for antigen capture. Importantly, target protein detections down to pg/ml concentrations were successfully demonstrated with a multiplexing capacity expanded to 11-plex. The oriented immobilization strategies performed best by providing better linear responses and more sensitive protein target detection. We believe that these results demonstrate the power of AFFIRM to be instrumental for verification of potential biomarkers present down to pg/ml concentrations at which thousands of tissue leakage proteins can be expected.

## Supporting information

S1 Table List of target proteins, peptides and scFv’s used in the AFFIRM assay for the set of 11 target proteins. (DOCX)

S2 Table Complete transition list for the 11 target proteins included in the study. (DOCX)

S3 Table Calculated percent coefficient of variation (CV), expressed as the percent relative standard deviation (standard deviation divided by the mean) for the best peptide (lowest detected concentration with a linear response) per target protein in the 11-plex assay. (DOCX)

S4 Table Proteins and corresponding peptides measured in the SRM assay evaluating on-bead versus in-solution antigen capture. (DOCX)

S1 Fig Measured intensities of peptides from target proteins from multiplexed captures in the streptavidin format. (DOCX)

S2 Fig Measured intensities of peptides from target proteins from multiplexed captures in the FLAG format. (DOCX)

S3 Fig Measured intensities of peptides from target proteins from multiplexed captures in the epoxy format. (DOCX)

S4 Fig Measured peptide intensities of scFv’s used in single and multiplexed captures using the three bead systems. (DOCX)

S5 Fig Measured peptide intensities of scFv’s used in the on-bead vs. in-solution experiments. (DOCX)

S6 Fig Signal intensity obtained for IL6 (A) and GAK (B) protein spiked in different backgrounds. 50 ng/ml of target protein was spiked in 10%, 20%, 50%, 80% plasma and 10% serum. F = Anti-FLAG plasma, SERUM = Anti-FLAG serum 10%, 1 and 2 –Duplicates. For IL6 (A) similar amounts of target protein was recovered independently of plasma concentration or serum. The same was seen for GAK protein (B) but with a trend of even increasingly concentrations of GAK protein recovery with increasing background concentration.
(DOCX)
