# Supplementary material for: Advancing the immunoaffinity platform AFFIRM to targeted measurements of proteins in serum in the pg/ml range
Source: PLoS One. 2018 Feb 13;13(2):e0189116. doi: 10.1371/journal.pone.0189116 (PMC5810979; doi:10.1371/journal.pone.0189116)
Supplement: S1 Table — (DOCX) [file pone.0189116.s001.docx]

S1 Table. List of target proteins, peptides and scFv’s used in the AFFIRM assay for the set of 11 target proteins.

| Protein | Protein ID | scFv | Peptide |
| --- | --- | --- | --- |
| Interleukin-6 (IL6) | P05231 | G-IL6-2 | YILDGISALR |
|  |  | G-IL6-8 | VLIQFLQK |
|  |  |  | NLDAITTPDPTTNASLLTK |
| Serine/threonine-protein phosphatase (PGAM5) | Q96HS1 | I-PGAM5-1 | EQAELTGLR |
|  |  | I-PGAM5-2 | AIETTDIISR |
|  |  |  | TLGDTGFMPPDK |
| Casein kinase I isoform epsilon (CSNK1E) | P49674 | L-CSNK1E-3 | TVLLLADQMISR |
|  |  | L-CSNK1E-5 | FDDKPDYSYLR |
| Alpha-1-syntrophin (SNTA1) | Q13424 | N-SNTA1-2 | ADAGGLGISIK |
|  |  | N-SNTA1-3 | EVVLEVK |
|  |  |  | NSTGGTSVGWDSPPASPLQR |
| Calcium/calmodulin-dependent protein kinase type IV (KCC4) | Q16566 | N-KCC4-1 | GATSIVYR |
|  |  |  | TEIGVLLR |
|  |  |  | IVEHQVLMK |
|  |  |  | LTTFQALQHPWVTGK |
| Serine/threonine-protein kinase (MARK2-1) | Q7KZI7 | N-MARK2-2 | ISGTSMAFK |
|  |  | N-MARK2-3 | TTSSMEPNEMMR |
| Tyrosine-protein kinase (FER) | P16591 | L-FER-2 | SDIVLLLSQK |
|  |  | L-FER-7 |  |
| Cyclin-G-associated kinase (GAK) | O14976 | H-GAK-20 | DQSDFVGQTVELGELR |
|  |  | H-GAK-27 | ALVEEEITR |
|  |  |  | AMLQVNPEER |
| InaD-like protein (INADL-1) | Q8NI35 | N-INADL-2 | NAGQVVHLTLVR |
|  |  |  | NDNIQALEK |
|  |  |  | DGQSLGIR |
|  |  |  | LLPIHTLR |
| Receptor-type tyrosine-protein phosphatase eta (PTPRJ) | Q12913 | I-PTPRJ-2 | VENFEAYFK |
|  |  | I-PTPRJ-8 | YAAELAENR |
|  |  |  | YNNVLPYDISR |
| Receptor-type tyrosine-protein phosphatase T (PTPRT) | O14522 | I-PTPRT-5 | YGNIISYDHSR |
|  |  |  | VTLIETEPLAEYVIR |
|  |  |  | VADLLQHITQMK |
|  |  |  | HYIATQGPMQETVK |
| Apolipoprotein B-100 (APOB) | P04114 |  | ITENDIQIALDDAK |
| All scFv’s |  |  | NTLYLQMNSLR |
