# Supplementary material for: Advancing the immunoaffinity platform AFFIRM to targeted measurements of proteins in serum in the pg/ml range
Source: PLoS One. 2018 Feb 13;13(2):e0189116. doi: 10.1371/journal.pone.0189116 (PMC5810979; doi:10.1371/journal.pone.0189116)
Supplement: S2 Table — (DOCX) [file pone.0189116.s002.docx]

S2 Table. Complete transition list for the 11 target proteins included in the study.

| Q1 mass | Q3 mass | Collision Energy | Peptide sequence | Protein name | Fragment Ion | Transition Ranking |
| --- | --- | --- | --- | --- | --- | --- |
| 560.821681 | 844.488693 | 19.7 | YILDGISALR | sp\|P05231\|IL6_HUMAN | y8 | 1 |
| 560.821681 | 731.404629 | 19.7 | YILDGISALR | sp\|P05231\|IL6_HUMAN | y7 | 2 |
| 560.821681 | 616.377686 | 19.7 | YILDGISALR | sp\|P05231\|IL6_HUMAN | y6 | 4 |
| 560.821681 | 446.272158 | 19.7 | YILDGISALR | sp\|P05231\|IL6_HUMAN | y4 | 3 |
| 494.813127 | 889.550565 | 17.7 | VLIQFLQK | sp\|P05231\|IL6_HUMAN | y7 | 5 |
| 494.813127 | 776.466501 | 17.7 | VLIQFLQK | sp\|P05231\|IL6_HUMAN | y6 | 1 |
| 494.813127 | 663.382437 | 17.7 | VLIQFLQK | sp\|P05231\|IL6_HUMAN | y5 | 2 |
| 494.813127 | 535.323859 | 17.7 | VLIQFLQK | sp\|P05231\|IL6_HUMAN | y4 | 4 |
| 993.523127 | 1459.763865 | 32.7 | NLDAITTPDPTTNASLLTK | sp\|P05231\|IL6_HUMAN | y14 | 2 |
| 993.523127 | 1358.716186 | 32.7 | NLDAITTPDPTTNASLLTK | sp\|P05231\|IL6_HUMAN | y13 | 3 |
| 993.523127 | 1257.668508 | 32.7 | NLDAITTPDPTTNASLLTK | sp\|P05231\|IL6_HUMAN | y12 | 1 |
| 993.523127 | 1045.588801 | 32.7 | NLDAITTPDPTTNASLLTK | sp\|P05231\|IL6_HUMAN | y10 | 4 |
| 508.772188 | 759.435929 | 18.2 | EQAELTGLR | sp\|Q96HS1\|PGAM5_HUMAN | y7 | 4 |
| 508.772188 | 688.398815 | 18.2 | EQAELTGLR | sp\|Q96HS1\|PGAM5_HUMAN | y6 | 6 |
| 508.772188 | 559.356222 | 18.2 | EQAELTGLR | sp\|Q96HS1\|PGAM5_HUMAN | y5 | 2 |
| 508.772188 | 446.272158 | 18.2 | EQAELTGLR | sp\|Q96HS1\|PGAM5_HUMAN | y4 | 1 |
| 559.806228 | 934.484001 | 19.7 | AIETTDIISR | sp\|Q96HS1\|PGAM5_HUMAN | y8 | 1 |
| 559.806228 | 805.441408 | 19.7 | AIETTDIISR | sp\|Q96HS1\|PGAM5_HUMAN | y7 | 2 |
| 559.806228 | 704.39373 | 19.7 | AIETTDIISR | sp\|Q96HS1\|PGAM5_HUMAN | y6 | 6 |
| 559.806228 | 603.346051 | 19.7 | AIETTDIISR | sp\|Q96HS1\|PGAM5_HUMAN | y5 | 3 |
| 639.80537 | 1064.471722 | 22.1 | TLGDTGFMPPDK | sp\|Q96HS1\|PGAM5_HUMAN | y10 | 2 |
| 639.80537 | 892.423315 | 22.1 | TLGDTGFMPPDK | sp\|Q96HS1\|PGAM5_HUMAN | y8 | 3 |
| 639.80537 | 791.375637 | 22.1 | TLGDTGFMPPDK | sp\|Q96HS1\|PGAM5_HUMAN | y7 | 4 |
| 639.80537 | 456.245275 | 22.1 | TLGDTGFMPPDK | sp\|Q96HS1\|PGAM5_HUMAN | y4 | 1 |
| 609.322676 | 955.557107 | 21.2 | MMQGGVGIPSIK | sp\|P49674\|KC1E_HUMAN | y10 | 5 |
| 609.322676 | 827.498529 | 21.2 | MMQGGVGIPSIK | sp\|P49674\|KC1E_HUMAN | y9 | 4 |
| 609.322676 | 614.387188 | 21.2 | MMQGGVGIPSIK | sp\|P49674\|KC1E_HUMAN | y6 | 6 |
| 609.322676 | 444.28166 | 21.2 | MMQGGVGIPSIK | sp\|P49674\|KC1E_HUMAN | y4 | 1 |
| 680.386862 | 1159.650355 | 23.3 | TVLLLADQMISR | sp\|P49674\|KC1E_HUMAN | y10 | 4 |
| 680.386862 | 933.482227 | 23.3 | TVLLLADQMISR | sp\|P49674\|KC1E_HUMAN | y8 | 1 |
| 680.386862 | 820.398163 | 23.3 | TVLLLADQMISR | sp\|P49674\|KC1E_HUMAN | y7 | 3 |
| 680.386862 | 749.361049 | 23.3 | TVLLLADQMISR | sp\|P49674\|KC1E_HUMAN | y6 | 5 |
| 709.832974 | 913.441408 | 24.2 | FDDKPDYSYLR | sp\|P49674\|KC1E_HUMAN | y7 | 1 |
| 709.832974 | 816.388644 | 24.2 | FDDKPDYSYLR | sp\|P49674\|KC1E_HUMAN | y6 | 4 |
| 709.832974 | 701.361701 | 24.2 | FDDKPDYSYLR | sp\|P49674\|KC1E_HUMAN | y5 | 2 |
| 709.832974 | 538.298373 | 24.2 | FDDKPDYSYLR | sp\|P49674\|KC1E_HUMAN | y4 | 3 |
| 501.284931 | 815.498529 | 17.9 | ADAGGLGISIK | sp\|Q13424\|SNTA1_HUMAN | y9 | 2 |
| 501.284931 | 744.461415 | 17.9 | ADAGGLGISIK | sp\|Q13424\|SNTA1_HUMAN | y8 | 4 |
| 501.284931 | 687.439952 | 17.9 | ADAGGLGISIK | sp\|Q13424\|SNTA1_HUMAN | y7 | 5 |
| 501.284931 | 630.418488 | 17.9 | ADAGGLGISIK | sp\|Q13424\|SNTA1_HUMAN | y6 | 6 |
| 501.284931 | 517.334424 | 17.9 | ADAGGLGISIK | sp\|Q13424\|SNTA1_HUMAN | y5 | 1 |
| 408.247286 | 587.376289 | 15.2 | EVVLEVK | sp\|Q13424\|SNTA1_HUMAN | y5 | 4 |
| 408.247286 | 488.307875 | 15.2 | EVVLEVK | sp\|Q13424\|SNTA1_HUMAN | y4 | 3 |
| 408.247286 | 375.223811 | 15.2 | EVVLEVK | sp\|Q13424\|SNTA1_HUMAN | y3 | 1 |
| 408.247286 | 246.144832 | 15.2 | EVVLEVK | sp\|Q13424\|SNTA1_HUMAN | c2 | 2 |
| 1007.484867 | 1310.648775 | 33.1 | NSTGGTSVGWDSPPASPLQR | sp\|Q13424\|SNTA1_HUMAN | y12 | 2 |
| 1007.484867 | 952.521056 | 33.1 | NSTGGTSVGWDSPPASPLQR | sp\|Q13424\|SNTA1_HUMAN | y9 | 3 |
| 1007.484867 | 865.489027 | 33.1 | NSTGGTSVGWDSPPASPLQR | sp\|Q13424\|SNTA1_HUMAN | y8 | 1 |
| 1007.484867 | 513.314357 | 33.1 | NSTGGTSVGWDSPPASPLQR | sp\|Q13424\|SNTA1_HUMAN | y4 | 4 |
| 433.740159 | 738.414465 | 15.9 | GATSIVYR | sp\|Q16566\|KCC4_HUMAN | y6 | 4 |
| 433.740159 | 637.366787 | 15.9 | GATSIVYR | sp\|Q16566\|KCC4_HUMAN | y5 | 3 |
| 433.740159 | 550.334758 | 15.9 | GATSIVYR | sp\|Q16566\|KCC4_HUMAN | y4 | 2 |
| 433.740159 | 437.250694 | 15.9 | GATSIVYR | sp\|Q16566\|KCC4_HUMAN | y3 | 6 |
| 450.779285 | 670.461021 | 16.4 | TEIGVLLR | sp\|Q16566\|KCC4_HUMAN | y6 | 1 |
| 450.779285 | 557.376957 | 16.4 | TEIGVLLR | sp\|Q16566\|KCC4_HUMAN | y5 | 2 |
| 450.779285 | 500.355494 | 16.4 | TEIGVLLR | sp\|Q16566\|KCC4_HUMAN | y4 | 4 |
| 450.779285 | 401.28708 | 16.4 | TEIGVLLR | sp\|Q16566\|KCC4_HUMAN | y3 | 3 |
| 548.812801 | 884.465849 | 19.4 | IVEHQVLMK | sp\|Q16566\|KCC4_HUMAN | y7 | 1 |
| 548.812801 | 755.423256 | 19.4 | IVEHQVLMK | sp\|Q16566\|KCC4_HUMAN | y6 | 2 |
| 548.812801 | 618.364344 | 19.4 | IVEHQVLMK | sp\|Q16566\|KCC4_HUMAN | y5 | 4 |
| 548.812801 | 490.305766 | 19.4 | IVEHQVLMK | sp\|Q16566\|KCC4_HUMAN | y4 | 6 |
| 863.967396 | 1136.621104 | 28.8 | LTTFQALQHPWVTGK | sp\|Q16566\|KCC4_HUMAN | y10 | 3 |
| 863.967396 | 1065.58399 | 28.8 | LTTFQALQHPWVTGK | sp\|Q16566\|KCC4_HUMAN | y9 | 4 |
| 863.967396 | 824.441349 | 28.8 | LTTFQALQHPWVTGK | sp\|Q16566\|KCC4_HUMAN | y7 | 2 |
| 863.967396 | 687.382437 | 28.8 | LTTFQALQHPWVTGK | sp\|Q16566\|KCC4_HUMAN | y6 | 1 |
| 707.293987 | 1211.48534 | 24.1 | TTSSMEPNEMMR | sp\|Q7KZI7\|MARK2_HUMAN | y10 | 3 |
| 707.293987 | 1037.421283 | 24.1 | TTSSMEPNEMMR | sp\|Q7KZI7\|MARK2_HUMAN | y8 | 4 |
| 707.293987 | 906.380799 | 24.1 | TTSSMEPNEMMR | sp\|Q7KZI7\|MARK2_HUMAN | y7 | 2 |
| 707.293987 | 777.338206 | 24.1 | TTSSMEPNEMMR | sp\|Q7KZI7\|MARK2_HUMAN | y6 | 1 |
| 471.241678 | 828.392015 | 17 | ISGTSMAFK | sp\|Q7KZI7\|MARK2_HUMAN | y8 | 2 |
| 471.241678 | 741.359987 | 17 | ISGTSMAFK | sp\|Q7KZI7\|MARK2_HUMAN | y7 | 1 |
| 471.241678 | 684.338523 | 17 | ISGTSMAFK | sp\|Q7KZI7\|MARK2_HUMAN | y6 | 4 |
| 471.241678 | 583.290845 | 17 | ISGTSMAFK | sp\|Q7KZI7\|MARK2_HUMAN | y5 | 3 |
| 723.857846 | 1137.564711 | 24.6 | THAEDLNSGPLHR | sp\|P16591\|FER_HUMAN | y10 | 5 |
| 723.857846 | 893.495175 | 24.6 | THAEDLNSGPLHR | sp\|P16591\|FER_HUMAN | y8 | 2 |
| 723.857846 | 780.411111 | 24.6 | THAEDLNSGPLHR | sp\|P16591\|FER_HUMAN | y7 | 4 |
| 723.857846 | 666.368184 | 24.6 | THAEDLNSGPLHR | sp\|P16591\|FER_HUMAN | y6 | 8 |
| 723.857846 | 579.336155 | 24.6 | THAEDLNSGPLHR | sp\|P16591\|FER_HUMAN | y5 | 7 |
| 723.857846 | 522.314692 | 24.6 | THAEDLNSGPLHR | sp\|P16591\|FER_HUMAN | y4 | 6 |
| 790.442503 | 1198.694269 | 26.6 | LHMLHNQYVLALK | sp\|P16591\|FER_HUMAN | y10 | 3 |
| 790.442503 | 1085.610205 | 26.6 | LHMLHNQYVLALK | sp\|P16591\|FER_HUMAN | y9 | 1 |
| 790.442503 | 948.551293 | 26.6 | LHMLHNQYVLALK | sp\|P16591\|FER_HUMAN | y8 | 4 |
| 790.442503 | 706.449788 | 26.6 | LHMLHNQYVLALK | sp\|P16591\|FER_HUMAN | y6 | 6 |
| 790.442503 | 543.38646 | 26.6 | LHMLHNQYVLALK | sp\|P16591\|FER_HUMAN | y5 | 5 |
| 558.337164 | 913.60808 | 19.7 | SDIVLLLSQK | sp\|P16591\|FER_HUMAN | y8 | 6 |
| 558.337164 | 800.524016 | 19.7 | SDIVLLLSQK | sp\|P16591\|FER_HUMAN | y7 | 5 |
| 558.337164 | 588.371538 | 19.7 | SDIVLLLSQK | sp\|P16591\|FER_HUMAN | y5 | 4 |
| 558.337164 | 475.287474 | 19.7 | SDIVLLLSQK | sp\|P16591\|FER_HUMAN | y4 | 2 |
| 896.93923 | 1101.589864 | 29.8 | DQSDFVGQTVELGELR | sp\|O14976\|GAK_HUMAN | y10 | 1 |
| 896.93923 | 916.509822 | 29.8 | DQSDFVGQTVELGELR | sp\|O14976\|GAK_HUMAN | y8 | 6 |
| 896.93923 | 716.39373 | 29.8 | DQSDFVGQTVELGELR | sp\|O14976\|GAK_HUMAN | y6 | 2 |
| 896.93923 | 474.267073 | 29.8 | DQSDFVGQTVELGELR | sp\|O14976\|GAK_HUMAN | y4 | 5 |
| 530.287671 | 875.446888 | 18.8 | ALVEEEITR | sp\|O14976\|GAK_HUMAN | y7 | 1 |
| 530.287671 | 776.378474 | 18.8 | ALVEEEITR | sp\|O14976\|GAK_HUMAN | y6 | 2 |
| 530.287671 | 647.335881 | 18.8 | ALVEEEITR | sp\|O14976\|GAK_HUMAN | y5 | 3 |
| 530.287671 | 518.293287 | 18.8 | ALVEEEITR | sp\|O14976\|GAK_HUMAN | y4 | 5 |
| 593.79788 | 871.426821 | 20.7 | AMLQVNPEER | sp\|O14976\|GAK_HUMAN | y7 | 4 |
| 593.79788 | 743.368243 | 20.7 | AMLQVNPEER | sp\|O14976\|GAK_HUMAN | y6 | 2 |
| 593.79788 | 644.299829 | 20.7 | AMLQVNPEER | sp\|O14976\|GAK_HUMAN | y5 | 1 |
| 593.79788 | 530.256902 | 20.7 | AMLQVNPEER | sp\|O14976\|GAK_HUMAN | y4 | 3 |
| 423.227416 | 673.399149 | 15.6 | DGQSLGIR | sp\|Q8NI35\|INADL_HUMAN | y6 | 6 |
| 423.227416 | 545.340572 | 15.6 | DGQSLGIR | sp\|Q8NI35\|INADL_HUMAN | y5 | 2 |
| 423.227416 | 458.308544 | 15.6 | DGQSLGIR | sp\|Q8NI35\|INADL_HUMAN | y4 | 3 |
| 423.227416 | 345.22448 | 15.6 | DGQSLGIR | sp\|Q8NI35\|INADL_HUMAN | y3 | 1 |
| 653.883135 | 936.598912 | 22.5 | NAGQVVHLTLVR | sp\|Q8NI35\|INADL_HUMAN | y8 | 2 |
| 653.883135 | 837.530498 | 22.5 | NAGQVVHLTLVR | sp\|Q8NI35\|INADL_HUMAN | y7 | 1 |
| 653.883135 | 738.462084 | 22.5 | NAGQVVHLTLVR | sp\|Q8NI35\|INADL_HUMAN | y6 | 3 |
| 653.883135 | 601.403172 | 22.5 | NAGQVVHLTLVR | sp\|Q8NI35\|INADL_HUMAN | y5 | 5 |
| 522.769645 | 815.462144 | 18.6 | NDNIQALEK | sp\|Q8NI35\|INADL_HUMAN | y7 | 2 |
| 522.769645 | 701.419216 | 18.6 | NDNIQALEK | sp\|Q8NI35\|INADL_HUMAN | y6 | 5 |
| 522.769645 | 588.335152 | 18.6 | NDNIQALEK | sp\|Q8NI35\|INADL_HUMAN | y5 | 1 |
| 522.769645 | 460.276575 | 18.6 | NDNIQALEK | sp\|Q8NI35\|INADL_HUMAN | y4 | 3 |
| 564.282585 | 1028.489481 | 19.8 | VPDSPENELK | sp\|Q8NI35\|INADL_HUMAN | y9 | 4 |
| 564.282585 | 931.436717 | 19.8 | VPDSPENELK | sp\|Q8NI35\|INADL_HUMAN | y8 | 1 |
| 564.282585 | 816.409774 | 19.8 | VPDSPENELK | sp\|Q8NI35\|INADL_HUMAN | y7 | 3 |
| 564.282585 | 729.377745 | 19.8 | VPDSPENELK | sp\|Q8NI35\|INADL_HUMAN | y6 | 2 |
| 564.282585 | 503.282388 | 19.8 | VPDSPENELK | sp\|Q8NI35\|INADL_HUMAN | y4 | 5 |
| 481.810919 | 736.446434 | 17.4 | LLPIHTLR | sp\|Q8NI35\|INADL_HUMAN | y6 | 1 |
| 481.810919 | 639.39367 | 17.4 | LLPIHTLR | sp\|Q8NI35\|INADL_HUMAN | y5 | 4 |
| 481.810919 | 526.309606 | 17.4 | LLPIHTLR | sp\|Q8NI35\|INADL_HUMAN | y4 | 2 |
| 481.810919 | 389.250694 | 17.4 | LLPIHTLR | sp\|Q8NI35\|INADL_HUMAN | y3 | 3 |
| 573.776939 | 918.435595 | 20.1 | VENFEAYFK | sp\|Q12913\|PTPRJ_HUMAN | y7 | 1 |
| 573.776939 | 804.392667 | 20.1 | VENFEAYFK | sp\|Q12913\|PTPRJ_HUMAN | y6 | 5 |
| 573.776939 | 657.324253 | 20.1 | VENFEAYFK | sp\|Q12913\|PTPRJ_HUMAN | y5 | 3 |
| 573.776939 | 528.28166 | 20.1 | VENFEAYFK | sp\|Q12913\|PTPRJ_HUMAN | y4 | 2 |
| 518.756538 | 802.405357 | 18.5 | YAAELAENR | sp\|Q12913\|PTPRJ_HUMAN | y7 | 1 |
| 518.756538 | 731.368243 | 18.5 | YAAELAENR | sp\|Q12913\|PTPRJ_HUMAN | y6 | 5 |
| 518.756538 | 602.32565 | 18.5 | YAAELAENR | sp\|Q12913\|PTPRJ_HUMAN | y5 | 3 |
| 518.756538 | 489.241586 | 18.5 | YAAELAENR | sp\|Q12913\|PTPRJ_HUMAN | y4 | 2 |
| 677.343509 | 1076.573485 | 23.2 | YNNVLPYDISR | sp\|Q12913\|PTPRJ_HUMAN | y9 | 3 |
| 677.343509 | 962.530558 | 23.2 | YNNVLPYDISR | sp\|Q12913\|PTPRJ_HUMAN | y8 | 4 |
| 677.343509 | 863.462144 | 23.2 | YNNVLPYDISR | sp\|Q12913\|PTPRJ_HUMAN | y7 | 1 |
| 677.343509 | 750.37808 | 23.2 | YNNVLPYDISR | sp\|Q12913\|PTPRJ_HUMAN | y6 | 2 |
| 698.884486 | 998.545162 | 23.9 | VADLLQHITQMK | sp\|O14522\|PTPRT_HUMAN | y8 | 3 |
| 698.884486 | 885.461098 | 23.9 | VADLLQHITQMK | sp\|O14522\|PTPRT_HUMAN | y7 | 6 |
| 698.884486 | 757.40252 | 23.9 | VADLLQHITQMK | sp\|O14522\|PTPRT_HUMAN | y6 | 2 |
| 698.884486 | 507.259544 | 23.9 | VADLLQHITQMK | sp\|O14522\|PTPRT_HUMAN | y4 | 1 |
| 662.817658 | 990.50032 | 22.8 | YGNIISYDHSR | sp\|O14522\|PTPRT_HUMAN | y8 | 6 |
| 662.817658 | 877.416256 | 22.8 | YGNIISYDHSR | sp\|O14522\|PTPRT_HUMAN | y7 | 2 |
| 662.817658 | 764.332192 | 22.8 | YGNIISYDHSR | sp\|O14522\|PTPRT_HUMAN | y6 | 1 |
| 662.817658 | 677.300164 | 22.8 | YGNIISYDHSR | sp\|O14522\|PTPRT_HUMAN | y5 | 4 |
| 801.900865 | 1302.672213 | 27 | HYIATQGPMQETVK | sp\|O14522\|PTPRT_HUMAN | y12 | 1 |
| 801.900865 | 1189.588149 | 27 | HYIATQGPMQETVK | sp\|O14522\|PTPRT_HUMAN | y11 | 3 |
| 801.900865 | 1118.551035 | 27 | HYIATQGPMQETVK | sp\|O14522\|PTPRT_HUMAN | y10 | 4 |
| 801.900865 | 1017.503357 | 27 | HYIATQGPMQETVK | sp\|O14522\|PTPRT_HUMAN | y9 | 6 |
| 801.900865 | 889.444779 | 27 | HYIATQGPMQETVK | sp\|O14522\|PTPRT_HUMAN | y8 | 2 |
| 801.900865 | 832.423315 | 27 | HYIATQGPMQETVK | sp\|O14522\|PTPRT_HUMAN | y7 | 5 |
| 873.487827 | 1319.684158 | 29.1 | VTLIETEPLAEYVIR | sp\|O14522\|PTPRT_HUMAN | y11 | 4 |
| 873.487827 | 1190.641565 | 29.1 | VTLIETEPLAEYVIR | sp\|O14522\|PTPRT_HUMAN | y10 | 2 |
| 873.487827 | 1089.593886 | 29.1 | VTLIETEPLAEYVIR | sp\|O14522\|PTPRT_HUMAN | y9 | 3 |
| 873.487827 | 550.334758 | 29.1 | VTLIETEPLAEYVIR | sp\|O14522\|PTPRT_HUMAN | y4 | 5 |
| 676.853186 | 1137.60849 | 23.2 | NTLYLQMNSLR | scFv_All | y9 |  |
| 676.853186 | 1024.524426 | 23.2 | NTLYLQMNSLR | scFv_All | y8 |  |
| 676.853186 | 861.461098 | 23.2 | NTLYLQMNSLR | scFv_All | y7 |  |
| 676.853186 | 748.377034 | 23.2 | NTLYLQMNSLR | scFv_All | y6 |  |
| 779.901585 | 873.467623 | 26.3 | ITENDIQIALDDAK | sp\|P04114\|APOB_HUMAN | y8 |  |
| 779.901585 | 745.409046 | 26.3 | ITENDIQIALDDAK | sp\|P04114\|APOB_HUMAN | y7 |  |
| 779.901585 | 632.324982 | 26.3 | ITENDIQIALDDAK | sp\|P04114\|APOB_HUMAN | y6 |  |
| 779.901585 | 561.287868 | 26.3 | ITENDIQIALDDAK | sp\|P04114\|APOB_HUMAN | y5 |  |
