# Supplementary material for: Advancing the immunoaffinity platform AFFIRM to targeted measurements of proteins in serum in the pg/ml range
Source: PLoS One. 2018 Feb 13;13(2):e0189116. doi: 10.1371/journal.pone.0189116 (PMC5810979; doi:10.1371/journal.pone.0189116)
Supplement: S3 Table — (DOCX) [file pone.0189116.s003.docx]

S3 Table. Calculated percent coefficient of variation (CV), expressed as the percent relative standard deviation (standard deviation divided by the mean) for the best peptide (lowest detected concentration with a linear response) per target protein in the 11-plex assay.

| **Protein** | **Best peptide** | **Concentration (ng/ml)** | **CV (%) Streptavidin** | **CV (%) FLAG** | **CV (%) Epoxy** | **Alternative best peptide** |
| --- | --- | --- | --- | --- | --- | --- |
| **IL6** | YILDGISALR | 0,1 | 0,210 |  |  |  |
|  |  | 0,5 | 0,119 | 0,324 |  |  |
|  |  | 1 |  | 0,859 |  |  |
|  |  | 5 | 0,061 | 0,131 | 0,127 |  |
|  |  | 12,5 | 0,102 | 0,207 | 0,268 |  |
|  |  | 25 | 0,068 | 0,193 | 0,421 |  |
|  |  | 50 | 0,235 | 0,394 | 0,086 |  |
|  |  | 100 |  | 0,174 | 0,361 |  |
|  |  | **Mean** | **0,132** | **0,326** | **0,253** |  |
| **PGAM5** | EQAELTGLR | 0,1 | 0,274 |  |  |  |
|  |  | 0,5 | 0,226 |  |  |  |
|  |  | 1 |  | 0,385 |  |  |
|  |  | 5 | 0,007 | 0,189 |  |  |
|  |  | 12,5 | 0,091 | 0,106 | 0,191 |  |
|  |  | 25 | 0,097 | 0,236 | 0,130 |  |
|  |  | 50 | 0,258 | 0,300 | 0,242 |  |
|  |  | 100 |  | 0,305 | 0,117 |  |
|  |  | **Mean** | **0,159** | **0,253** | **0,170** |  |
| **CSNK1E** | TVLLLADQMISR | 5 | 0,566 |  |  |  |
|  |  | 12,5 | 0,258 |  |  |  |
|  |  | 25 | 0,233 |  |  |  |
|  |  | 50 | 0,186 |  |  |  |
|  |  | 100 |  |  |  |  |
|  |  | **Mean** | **0,311** |  |  |  |
| **SNTA1** | EVVLEVK | 0,05 | 0,241 |  |  |  |
|  |  | 0,1 | 0,368 |  |  |  |
|  |  | 0,5 | 0,009 | 0,397 |  |  |
|  |  | 1 |  | 0,496 |  |  |
|  |  | 5 | 0,088 | 0,319 |  |  |
|  |  | 12,5 | 0,059 | 0,275 |  |  |
|  |  | 25 | 0,080 | 0,134 |  |  |
|  |  | 50 | 0,322 | 0,457 |  |  |
|  |  | 100 |  | 0,190 |  |  |
|  |  | **Mean** | **0,154** | **0,324** |  |  |
| **KCC4** | TEIGVLLR |  |  |  |  |  |
|  |  | 0,5 | 0,085 |  |  |  |
|  |  | 1 |  | 0,280 |  |  |
|  |  | 5 | 0,051 | 0,114 |  |  |
|  |  | 12,5 | 0,148 | 0,268 | 0,350 |  |
|  |  | 25 | 0,298 | 0,214 | 0,890 |  |
|  |  | 50 | 0,276 | 0,203 | 0,334 |  |
|  |  | 100 |  | 0,288 | 0,517 |  |
|  |  | **Mean** | **0,171** | **0,228** | **0,523** |  |
| **MARK2-1** | ISGTSMAFK | 0,05 | 0,720 |  |  |  |
|  |  | 0,1 | 0,473 |  |  |  |
|  |  | 0,5 | 0,285 |  |  |  |
|  |  | 1 |  | 0,703 | 0,183 | TTSSMEPNEMMR |
|  |  | 5 | 0,677 | 0,322 | 0,154 |  |
|  |  | 12,5 | 0,108 | 0,157 | 0,247 |  |
|  |  | 25 | 0,395 | 0,246 | 0,493 |  |
|  |  | 50 | 0,434 | 0,371 | 0,298 |  |
|  |  | 100 |  | 0,334 | 0,087 |  |
|  |  | **Mean** | **0,395** | **0,356** | **0,244** |  |
| **FER** | SDIVLLLSQK | 1 |  |  |  |  |
|  |  | 5 | 0,192 | 0,147 |  |  |
|  |  | 12,5 | 0,001 | 0,268 |  |  |
|  |  | 25 | 0,104 | 0,325 |  |  |
|  |  | 50 | 0,171 | 0,098 |  |  |
|  |  | 100 |  | 0,131 |  |  |
|  |  | **Mean** | **0,117** | **0,194** |  |  |
| **GAK** | DQSDFVGQTVELGELR | 0,5 | 0,237 | 0,057 |  |  |
|  |  | 1 |  | 0,393 |  |  |
|  |  | 5 | 0,049 | 0,102 |  |  |
|  |  | 12,5 | 0,076 | 0,117 | 1,217 |  |
|  |  | 25 | 0,192 | 0,224 | 0,572 |  |
|  |  | 50 | 0,223 | 0,087 | 0,307 |  |
|  |  | 100 |  | 0,238 | 0,669 |  |
|  |  | **Mean** | **0,155** | **0,174** | **0,691** |  |
| **INADL-1** | NDNIQALEK | 5 | 0,073 |  |  |  |
|  |  | 12,5 | 0,210 |  |  |  |
|  |  | 25 | 0,058 | 0,762 |  | NAGQVVHLTLVR |
|  |  | 50 | 0,277 | 0,196 |  |  |
|  |  | 100 |  | 0,455 |  |  |
|  |  | **Mean** | **0,154** | **0,471** |  |  |
| **PTPRJ** | YAAELAENR | 1 |  | 0,317 |  |  |
|  |  | 5 |  | 0,086 |  |  |
|  |  | 12,5 | 0,197 | 0,234 |  |  |
|  |  | 25 | 0,377 | 0,241 |  |  |
|  |  | 50 | 0,255 | 0,293 |  |  |
|  |  | 100 |  | 0,384 |  |  |
|  |  | **Mean** | **0,276** | **0,259** |  |  |
| **PTPRT** | VTLIETEPLAEYVIR | 5 | 0,062 | 0,172 |  |  |
|  |  | 12,5 | 0,113 | 0,291 | 0,500 |  |
|  |  | 25 | 0,418 | 0,294 | 0,389 |  |
|  |  | 50 | 0,259 | 0,369 | 0,509 |  |
|  |  | 100 |  | 0,257 | 0,136 |  |
|  |  | **Mean** | **0,213** | **0,277** | **0,383** |  |
