# Supplementary material for: Advancing the immunoaffinity platform AFFIRM to targeted measurements of proteins in serum in the pg/ml range
Source: PLoS One. 2018 Feb 13;13(2):e0189116. doi: 10.1371/journal.pone.0189116 (PMC5810979; doi:10.1371/journal.pone.0189116)
Supplement: S4 Table — (DOCX) [file pone.0189116.s004.docx]

S4 Table. Proteins and corresponding peptides measured in the SRM assay evaluating on-bead versus in-solution antigen capture.

| **Protein Name** | **Capture antibody** | **Protein ID** | **Target peptide sequence*** |
| --- | --- | --- | --- |
| Keratin, type I cytoskeletal 19 (Ker-19) | C-KER19-1 C-KER19-3 | P08727 | **FGPGVAFR LTMQNLNDR ILGATIENSR** |
| Phosphatidylinositol 3-kinase regulatory subunit alpha (p85A) | C-P85A-4 C-P85A-7 | P27986 | **DTADGTFLVR NESLAQYNPK LLYPVSK** |
| Apolipoprotein B-100 (APOB) |  | P04114 | **ITENDIQIALDDAK** |
| Complement C3 (CO3) |  | P01024 | FVTVQATFGTQVVEK  **TVMVNIENPEGIPVK** |
| Complement C5 (CO5) |  | P01031 | **FQNSAILTIQPK** |
| Heparin cofactor 2 (HEP2) |  | P05546 | EYYFAEAQIADFSDPAFISK  **TLEAQLTPR** |
| Serum albumin (ALBU) |  | P02768 | **AVMDDFAAFVEK** |
| Serotransferrin (TRFE) |  | P02787 | **MYLGYEYVTAIR** |
| Ceruloplasmin (CERU) |  | P00450 | **GPEEEHLGILGPVIWAEVGDTIR** |
| Gelsolin (GELS) |  | P06396 | **TPSAAYLWVGTGASEAEK** |
| Complement C4-A (CO4A) |  | P0C0L4 | **VDFTLSSER**  DFALLSLQVPLK |
| Complement C4-B (CO4B) |  | P0C0L5 | VDFTLSSER  **ASAGLLGAHAAAITAYALTLTK** |
| Haptoglobin (HPT) |  | P00738 | TEGDGVYTLNNEK  **VTSIQDWVQK** |
| scFv C-KER19-1 scFv C-KER19-3 scFv C-P85A-4 scFv C-P85A-7 |  |  | **NTLYLQMNSLR**  AEDTAVYYCAR |

* Peptides in bold are displayed in the result figures
