# Supplementary material for: Advancing the immunoaffinity platform AFFIRM to targeted measurements of proteins in serum in the pg/ml range
Source: PLoS One. 2018 Feb 13;13(2):e0189116. doi: 10.1371/journal.pone.0189116 (PMC5810979; doi:10.1371/journal.pone.0189116)
Supplement: S3 Fig — (DOCX) [file pone.0189116.s007.docx]

S3 Figure. Measured intensities of peptides from target proteins from multiplexed captures in the epoxy format.
