# Supplementary material for: Advancing the immunoaffinity platform AFFIRM to targeted measurements of proteins in serum in the pg/ml range
Source: PLoS One. 2018 Feb 13;13(2):e0189116. doi: 10.1371/journal.pone.0189116 (PMC5810979; doi:10.1371/journal.pone.0189116)
Supplement: S5 Fig — (DOCX) [file pone.0189116.s009.docx]

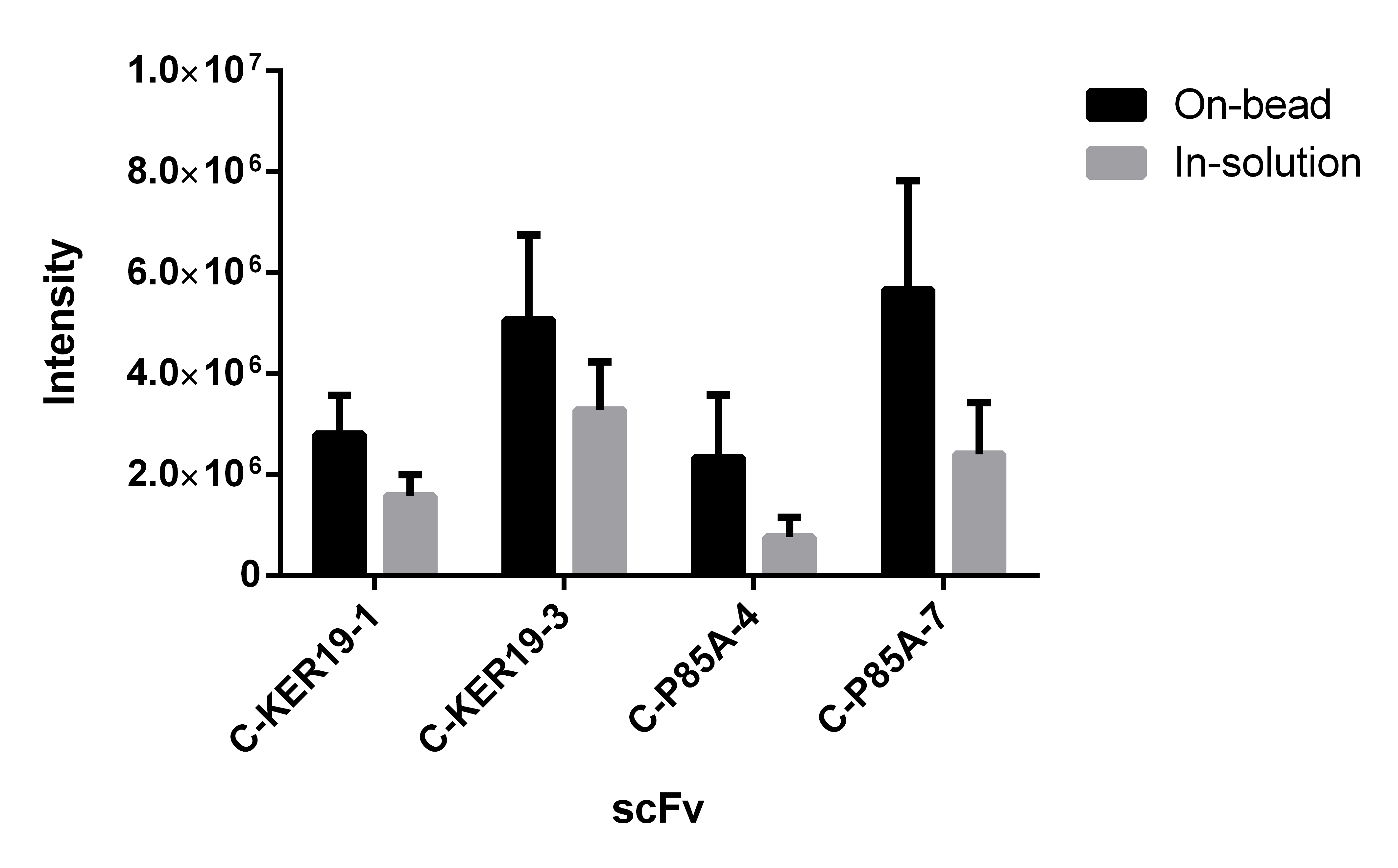


S5 Figure. Measured intensity of the scFv peptide NTLYLQMNSLR common to all four scFv C-KER19-1, C-KER19-3, C-P85A-4 and C-P85A-7 used in the on-bead vs. in-solution experiments. Comparing on-bead (black) and in-solution (grey) scFv peptide signals from captures.
