# Supplementary material for: Advancing the immunoaffinity platform AFFIRM to targeted measurements of proteins in serum in the pg/ml range
Source: PLoS One. 2018 Feb 13;13(2):e0189116. doi: 10.1371/journal.pone.0189116 (PMC5810979; doi:10.1371/journal.pone.0189116)
Supplement: S6 Fig — 50 ng/ml of target protein was spiked in 10%, 20%, 50%, 80% plasma and 10% serum. F = Anti-FLAG plasma, SERUM = Anti-FLAG serum 10%, 1 and 2 –Duplicates. For IL6 (A) similar amounts of target protein was recovered independently of plasma concentration or serum. The same was seen for GAK protein (B) but with a trend of even increasingly concentrations of GAK protein recovery with increasing background concentration. (DOCX) [file pone.0189116.s010.docx]

A

B

S6 Figure. Signal intensity obtained for IL6 (A) and GAK (B) protein spiked in different backgrounds. 50 ng/ml of target protein was spiked in 10%, 20%, 50%, 80% plasma and 10% serum. F = Anti-FLAG plasma, SERUM = Anti-FLAG serum 10%, 1 and 2 – Duplicates. For IL6 (A) similar amounts of target protein was recovered independently of plasma concentration or serum. The same was seen for GAK protein (B) but with a trend of even increasingly concentrations of GAK protein recovery with increasing background concentration.
